# Development, Validation of LC-MS/MS Method and Determination of Pharmacokinetic Parameters of the Stroke Neuroprotectant Neurounina-1 in Beagle Dog Plasma After Intravenous Administration

**DOI:** 10.3389/fphar.2019.00432

**Published:** 2019-04-25

**Authors:** Beatrice Severino, Angela Corvino, Ferdinando Fiorino, Francesco Frecentese, Elisa Perissutti, Giuseppe Caliendo, Vincenzo Santagada, Elisa Magli, Pasquale Molinaro, Giuseppe Pignataro, Lucio Annunziato, Natalícia J. Antunes, Julio Rojas-Moscoso, Noedi L. de Freitas, Gustavo D. Mendes, Gilberto De Nucci

**Affiliations:** ^1^Department of Pharmacy, School of Medicine, University of Naples Federico II, Naples, Italy; ^2^Department of Neuroscience, Reproductive and Odontostomatological Sciences, School of Medicine, University of Naples Federico II, Naples, Italy; ^3^IRCCS SDN, Naples, Italy; ^4^Department of Pharmacology, Faculty of Medical Sciences, State University of Campinas, Campinas, Brazil; ^5^Department of Pharmacology, Faculty of Medical Sciences, Metropolitan University of Santos, Santos, Brazil; ^6^Department of Pharmacology, Faculty of Medicine, São Leopoldo Mandic, Campinas, Brazil; ^7^Faculty of Medicine, University of Mogi das Cruzes, Mogi das Cruzes, Brazil; ^8^Department of Pharmacology, Faculty of Medical Sciences, University of São Paulo, São Paulo, Brazil

**Keywords:** neurounina-1, Na^+^/Ca^2+^ exchanger (NCX), LC-MS/MS, beagle dog plasma, pharmacokinetics

## Abstract

Neurounina-1 [chemical name: 7-nitro-5-phenyl-1-(pyrrolidin-1-ylmethyl)-1H-benzo[e][1,4]diazepin-2(3H)-one] is a new compound provided with relevant neuroprotective effect during stroke and in neonatal hypoxia by increasing the Na^+^/Ca^2+^ exchanger (NCX) isoforms NCX1 and NCX2 activity. This study shows for the first time, the development and validation of a sensitive and selective method for analysis of neurounina-1 in beagle dog plasma by liquid chromatography coupled to tandem mass spectrometry (LC-MS/MS). The sample preparation consisted of extraction of the analyte and the internal standard (IS) (ropivacaine) from plasma (50 μL) by liquid-liquid extraction using acetonitrile (100 μL). The selected reaction monitoring mode of the positive ion was performed and the precursor to the product ion transitions of m/z 365 > 83 and m/z 275 > 126 were used to measure the derivative of neurounina-1 and ropivacaine. The chromatographic separation was achieved using a Phenomenex C18 Luna (150 mm × 4.6 mm × 5 μm) analytical column with an isocratic mobile phase composed of methanol/acetonitrile/water (50/40/10, v/v/v) + 0.1% formic acid + 1 M ammonium formate. The method was linear over a concentration range of 1–500 ng/mL. The method was applied to evaluate the pharmacokinetics of neurounina-1 after a single intravenous administration of three different doses (0.1 mg/kg, 0.3 mg/kg, and 1 mg/kg) to beagle dogs (*n* = 5). The mean AUC_0-tlast_ values were 26.10, 115.81, and 257.28 ng^∗^h/mL following intravenous administration of 0.1, 0.3, and 1 mg/kg, respectively. Linear pharmacokinetics was observed up to 1.0 mg/kg. The neurounina-1 was rapidly eliminated, with mean CL values of 46.24, 47.57, and 69.15 L/h, Vd of 130.31, 154.15, and 210.79 L and t_1/2_ of 2.14, 2.54, and 2.04 h after intravenous administration of 0.1, 0.3, and 1 mg/kg, respectively. This new analytical method allows the rapid determination of the neurounina-1, a new developed compound, able to exert a remarkable neuroprotective effect in the low nanomolar range.

## Introduction

Stroke is a leading cause of long-lasting injury, disability, and death. Early treatment and preventive measures can reduce the brain damage that occurs as a result of a stroke. Several studies have demonstrated that the activation of the three Na^+^/Ca^2+^ exchanger (NCX) isoforms, NCX1, NCX2, and NCX3 exerts a neuroprotective action against stroke injury in adult mice (Pignataro et al., 2004a,b; Jeon et al., 2008; Molinaro et al., 2008, 2016) and against neonatal hypoxic-ischemic encephalopathy damage (Cerullo et al., 2018). Therefore, in recent years, the interest has been focused on the identification of NCX activator aiming to have new therapeutic tools able to limit the extension of ischemic brain damage (Annunziato et al., 2004). With this purpose, the structure of one of the most potent inhibitors, SM-15811, was modified obtaining 7-nitro-5-phenyl-1-(pyrrolidin-1-ylmethyl)-1H-benzo[e][1,4]diazepin-2(3H)-one, a new small molecule named neurounina-1 that was patented in 2012 (Pignataro et al., 2012). Several pharmacological experiments have demonstrated that this compound exerts a remarkable neuroprotective effect during stroke by stimulation of NCX1 and NCX2 activities (Molinaro et al., 2013). Neurounina-1 has a wide therapeutic window, as shown in an *in vivo* mouse model of transient middle cerebral artery occlusion (tMCAO). When intraperitoneally administered at doses of 0.003 to 30 μg/kg to mouse, neurounina-1 effect was notable even 5 h after ischemia induction, unlike other drugs used to date for treating stroke, which require a much earlier administration. Furthermore, neurounina-1 presents a high lipophilicity index and low toxicity (Molinaro et al., 2013).

To support pre-clinical studies with neurounina-1, the development of a bioanalytical method to monitor its concentration in biological matrices is required. There is no available method for neurounina-1 quantification. Moreover, its half maximal effective concentration (EC50) is in the picomolar to low nanomolar range (Molinaro et al., 2013), which requires a low dose administration and consequently, a sensitive and selective method of quantification.

This study presents for the first time, the development and validation of a sensitive and selective method to quantify neurounina-1 in beagle dog plasma using liquid chromatography coupled to tandem mass spectrometry (LC-MS/MS), with ropivacaine as internal standard (IS). The method showed a lower limit of quantification (LLOQ) of 1.0 ng/mL, which was used to evaluate the pharmacokinetics in plasma after a single intravenous administration of three different doses of neurounina-1 (0.1 mg/kg, 0.3 mg/kg, and 1 mg/kg) to beagle dogs (*n* = 5).

## Materials and Methods

### Chemical and Reagents

Ropivacaine was utilized as IS and was supplied by the United States Pharmacopeia (Rockville, MD, United States). Neurounina-1 was synthesized in house according to the previously reported study (Molinaro et al., 2013). Acetonitrile and methanol (HPLC grade) were purchased from J.T Baker (Phillipsburg, NJ, United States); ammonium acetate and hexane (analysis grade) from J.T Baker (Ecatepec, Mexico); ethyl ether (analysis grade) from Mallinckrodt (Phillipsburg, NJ, United States). The water was obtained from the purification system Synergy UV^®^ (Millipore, Molsheim, France).

### Calibration Standards and Quality Control

Stock solutions of neurounina-1 and ropivacaine were prepared in methanol/water (50/50, v/v). Calibration curves for neurounina-1 were prepared by adding the compound to blank plasma to yield final concentrations of 1, 2, 10, 50, 100, 200, 350, and 500 ng/mL. The calibration curves were performed in duplicate for each day’s assays. The quality control (QC) samples were prepared in blank plasma at the concentrations of 1, 1.5, 30, 240, and 400 ng/mL, respectively. For each validation, seven replicates were analyzed for each CQ level (three validations were performed). The spiked plasma samples (standards and QC) were extracted in each analytical batch along with the unknown samples.

### Sample Preparation

Aliquots (0.05 mL) of each plasma sample were added to glass tubes followed by 0.05 mL of IS (ropivacaine 100 ng/mL). The tubes were vortexed for 5 s and 0.1 mL of acetonitrile were added. The samples were vortexed for 40 s, again, and then centrifuged at 2000 ×*g* for 5 min. The obtained supernatants were transferred to microvials for analysis.

### Instrumentation and Software

#### Liquid Chromatography

Neuronina-1 was separated in a 150 mm × 4.6 mm x 5 μm Phenomenex C18Luna reversed phase analytical column (Phenomenex Inc., CA, United States). The temperature of the column was maintained constant at 65°C. The mobile phase used was composed by methanol/acetonitrile/water (50/40/10, v/v/v) + 0.1% of formic acid + 1 M ammonium formate at a flow rate of 1 mL/min (split 1:3). The auto-sampler was maintained at room temperature.

#### Mass Spectrometry

**FIGURE 1:**
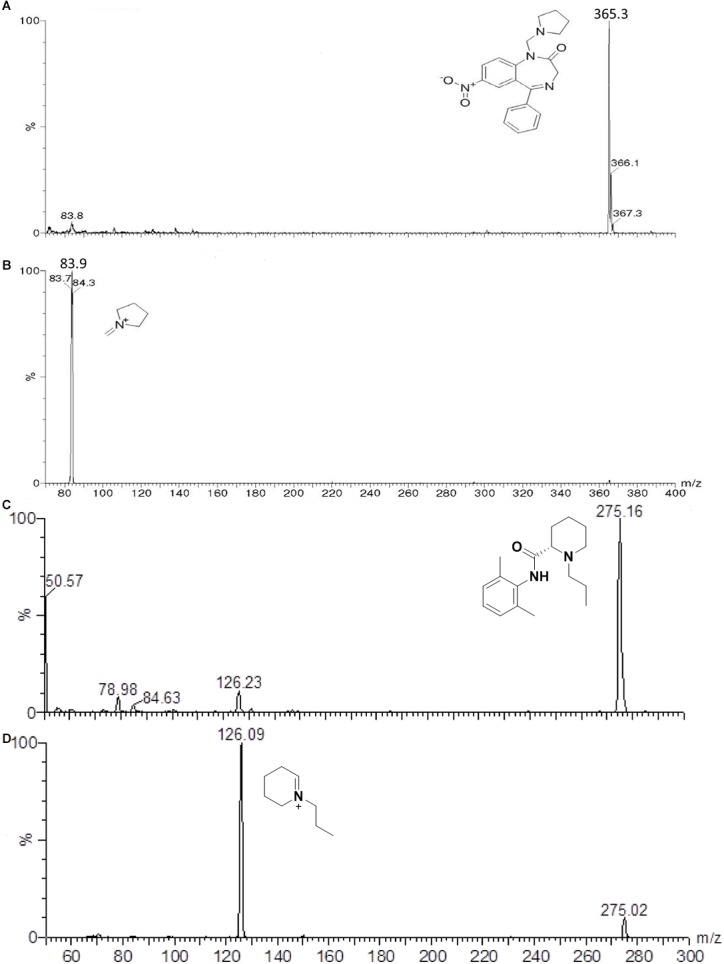
Full scan mass spectrum of the protonated molecular ion of neurounina-1 – m/z = 365.3 **(A)**, ion product of neurounina-1 – m/z = 83.9 **(B)**, protonated molecular ion of ropivacaine – m/z = 275.16 **(C)**, and the ion product of ropivacaine – m/z = 126.09 **(D)**.

The mass spectrometer (Quattro Micro – Micromass, Waters, United Kingdom) equipped with an electrospray source in the ESI positive polarity mode (ES+) was configured for multiple reaction monitoring (MRM) to monitor the transitions 365.3 > 83.9 and 275.2 > 126.0, for neurounina-1 and ropivacaine, respectively. Figure 1 shows the full scan spectra (upper trace) and the product ion spectra (lower trace) obtained by the proposed fragmentation pathways for neurounina-1 (panel A) and ropivacaine (panel B). To optimize all MS parameters, a standard solution of the analyte and IS was infused into the mass spectrometer. The optimized values of ion spray voltage, collision energy, and cone voltage were, respectively 2800 (V), 20 (eV), and 20 (V) for neurounina-1 and 2800 (V), 15 (eV), and 15 (V) for ropivacaine. Data acquisition and analysis were performed using the software Masslynx 4.0 (Waters Corporation, Milford, MA, United States).

### Method Validation

The method validation was carried out according to the United States Food and Drug Administration (FDA, 2001) bioanalytical method validation guidance and the Brazilian National Sanitary Surveillance Agency (Agência Nacional de Vigilância Sanitária [ANVISA], 2003).

#### Linearity

Calibration curves were prepared by assaying standard plasma samples at eight concentrations of neurounina-1 (1, 2, 10, 50, 100, 200, 350, and 500 ng/mL) and the linearity of each calibration curve was determined by plotting the peak area ratio (y) of neurounina-1/ropivacaine vs. nominal concentration of analyte. The calibration curve was constructed by weighted (1/X) least squares linear regression.

#### Accuracy and Precision

The precision and accuracy of assay was determined at five different concentrations (1.0, 1.5, 30, 240, and 400 ng/mL), selected based on the literature and comparison with the previously established analytical values for similar studies. The following criteria were found to approve precision and intra-race accuracy: (i) for each concentration level, coefficient of variation (CV) that does not exceed 15% for LLOQ and 20% for QC samples; (ii) mean value of the samples at each concentration level, within 85 – 115% of the actual value for LLOQ and 80–120% for QC samples.

#### Recovery and Matrix Effect

The recovery was evaluated by dividing the extracted sample mean response by the unextracted (spiked blank plasma extract) sample mean of the corresponding concentration. The matrix effect experiments were carried out using the ratio between spiked mobile phase solutions and unextracted samples, spiked on plasma residues.

#### Stability

To assess stability, QC plasma samples (1.5 and 400.0 ng/mL) were subjected to short-term (6 h) incubation at room temperature; four freeze/thaw (-20°C) cycles and 52 h in the autosampler at room temperature. Subsequently, the neurounina-1 concentrations were measured and compared with freshly prepared samples.

### Animals, Drug Administration and Blood Sampling

Beagle dogs (*n* = 5) of both sexes, aged between 2 and 3 years old, weighing between 10 and 14 kg were provided by Camilo Castelo Branco University, Brazil. The dogs were housed in Domingos Alves Veterinary Hospital in individual rooms and fed with a standard canine food. They had free access to water. The dogs underwent rigorous veterinary control and were all considered healthy based on physical examination and laboratory analysis performed before initiation and after completion of each occasion of the study. At the end of the study, the animals were returned to their habitat. This study was carried out in accordance with the recommendations of the general ethical guidelines established by the Brazilian Society for Laboratory Animal Science (SBCAL). The protocol was approved by the Committee for Ethics in Animal Use – State University of Campinas (CEUA/UNICAMP, protocol n° 3340-1).

After an overnight (8 h) fasting, a single intravenous dose of 0.1 mg/kg, 0.3 mg/kg, or 1 mg/kg neurounina-1 formulation was administered to beagle dogs, with 7 days washout period between each dose administration. Blood samples (5 mL) from a suitable antecubital vein were collected into heparin-containing tubes before and 0.03, 0.08, 0.17, 0.25, 0.33, 0.5, 1, 2, 3, 4, 6, 8, 12, and 24 h after the administration of each dose. A total of 210 mL of blood was collected during the study. The blood samples treated with sodium heparin were centrifuged at approximately 2000 ×*g* for 10 min at 4°C and the plasma was stored at -20°C until analysis.

### Pharmacokinetic and Statistical Analysis

Non-compartmental analysis was used to determine the pharmacokinetic parameters of neurounina-1 after the intravenous administration. The concentration at time zero (C_0_) was estimated by back-extrapolating from the elimination curve. The area under the plasma concentration vs. time curves from zero to the last detectable concentration (AUC_0-tlast_) were calculated by applying the linear-log trapezoid rule. Extrapolation of these areas to infinity (AUC_0-inf_) was done by adding the value C_last_/ke to the calculated AUC_0-tlast_ (where C_last_ = the last detectable concentration). Clearance (CL) was calculated by the formula dose/AUC_0-inf_. Volume of distribution (Vd) was calculated by the formula CL/ke. The WinNonlin software, version 6.4 (Pharsight Corp, Mountain View, CA, United States) was used. Statistical analysis was performed using GraphPad Prism software, version 3.2 (GraphPad Software, San Diego, California, United States).

## Results

### LC-MS/MS Determination

**FIGURE 2:**
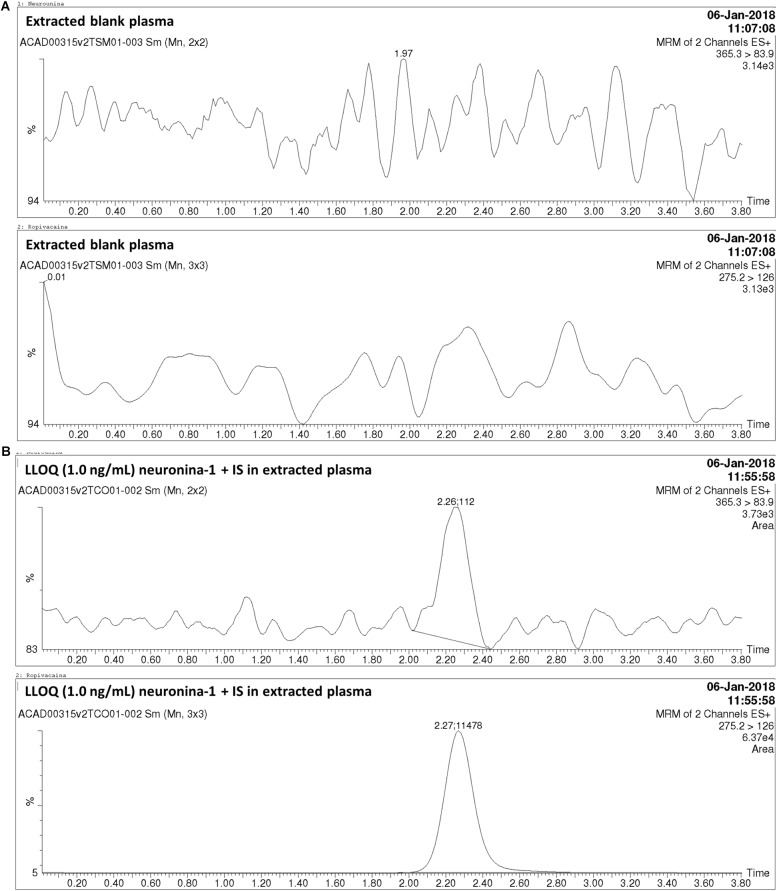
Chromatograms obtained in the analysis of neurounina-1 in beagle dog plasma. **(A)** Blank beagle dog plasma; **(B)** Blank beagle dog plasma spiked with neurounina-1 (lower limit of quantification (LLOQ – 1.0 ng/mL) and ropivacaine (internal standard – IS, 100 ng/mL).

Direct infusion was used to determine the best mass spectrometry conditions for neurounina-1 and ropivacaine. The spectrum for neurounina-1 showed a protonated molecular ion at m/z 365.3 and its collision-induced dissociation formed a distinctive product at m/z 83.9, corresponding to the 1-methylenepyrrolidinium ion. Ropivacaine showed a base peak ion ([M + H]^+^) at mass-to-charge ratio (m/z) of 275.16 and a fragmentation product at m/z 126.09, corresponding to the 1-propyl-2,3,4,5-tetrahydropyridinium ion (Figure 1). The selected reaction monitoring is based on the m/z 365.3 > 83.9 and 275.2 > 126.0 transitions, for neurounina-1 and ropivacaine, respectively. The total run time was 3.8 min (Figure 2). Ropivacaine was selected as the IS because of its behavior and structural similarity with neurounina-1.

The method was linear regression for neurounina-1 concentrations from 1 to 500 ng/mL (calibration curve *y* = 0.00767312x + 0.00170099, *r* = 0.997172). A linear regression with a weighting index of 1/x was performed on the peak area ratios of neurounina-1 and the IS vs. neurounina-1 concentrations of the eight beagle dogs plasma standards (in duplicate) to generate a calibration curve. The LLOQ, defined as the lowest concentration at which both the precision and accuracy were <20%, was 1.0 ng/mL. No endogenous peak was observed in the mass chromatogram of blank plasma (Figure 2). The retention times for neurounina-1 and IS were 2.27 min and 2.26 min, respectively (Figure 2).

### Reproducibility of the Method

The within- and between-run precision and accuracy for the LLOQ and QCs, summarized in Table 1, insure the reproducibility and repeatability of the results.

**Table 1:** Accuracy and precision data for neurounina-1 from the pre-study validation in beagle dog plasma.

Nominal value	1 ng/mL			1.5 ng/mL			30 ng/mL			240 ng/mL			400 ng/mL		
Batch identification	Val. 1	Val. 2	Val. 3	Val. 1	Val. 2	Val. 3	Val. 1	Val. 2	Val. 3	Val. 1	Val. 2	Val. 3	Val. 1	Val. 2	Val. 3
Individual concentration values	1.3	1.05	1.26	1.75	1.66	1.74	30.75	29.02	29.03	225.83	230.3	224.19	390.3	401.4	384.91
	1.13	1.01	0.88	1.52	1.72	1.46	30.78	29.01	28.68	228.15	237.39	227.31	393.52	400.99	383.35
	1.15	0.86	1.17	1.41	1.64	1.66	30.41	29.08	30.16	224.93	230.01	231.55	399.52	386.85	376.37
	1.21	1.04	0.81	1.52	1.28	1.55	29.84	30.96	29.35	228.29	229.99	225.94	386.95	392.3	382.44
	1.19	1.1	1.08	1.32	1.68	1.59	29.13	31.31	29.74	229	232.18	230.03	404.31	404.53	377.86
	1.22	1.11	1.09	1.71	1.8	1.6	28.71	28.45	29.71	234.66	232.4	226.96	392.94	389.26	374.23
	0.98	1.05	0.88	1.65	1.48	1.68	28.81	29.49	27.89	226.92	226.12	228.19	387.97	397.01	375.87
Mean (ng/mL)	1.17	1.03	1.02	1.55	1.61	1.61	29.8	29.6	29.2	228	231	228	394	396	379
Intra-run precision (CV%)	8.5	8.1	16.5	10.2	10.8	5.7	3.0	3.7	2.6	1.4	1.5	1.1	1.6	1.7	1.1
Intra-run accuracy (%)	116.9	103.1	102.4	103.6	107.2	107.4	99.3	98.7	97.4	95.1	96.3	94.9	98.4	99.0	94.8
Inter-run mean (ng/mL)	1.075			1.59			29.54			229.1			389.7		
Inter-run precision (CV%)	12.6			8.9			3.1			1.4			2.4		
Inter-run accuracy (%)	107.5			106.1			98.5			95.4			97.4		


*Val, validation; CV% = [(SD/M) × 100]; Accuracy % = [(E - T)/T] × 100; CV, coefficient of variation; M, mean; SD, standard deviation of M; E, experimentally determined concentration; T, theoretical concentration.*

The recovery obtained with the method of extraction of neurounina-1 with acetonitrile was higher than 89%. The matrix effect was practically absent in the analysis of neurounina-1 in beagle dog plasma (Table 2). Values >91% were obtained when the peak areas resulting from the injection of standard solutions in the mobile phase were compared with those of standard solutions added to extracts of blank plasma from eight different lots.

**Table 2:** Recovery and matrix effect for neurounina-1 in eight different lots of beagle dog plasma (4 normal, 2 lipemic, and 2 hemolysate).

Neurounina-1 concentration	1.5 ng/mL			400 ng/mL		
	Standard solution	Spiked blank plasma extract sample	Extracted sample	Standard solution	Spiked blank plasma extract sample	Extracted sample
Individual peak area values	123.5	133.8	125.0	32951	31780.1	29320.2
	107.3	131.9	106.9	32867.8	32355.4	31935.9
	145.2	126.5	120.7	33273.9	34053.7	29296.7
Mean	125.3	130.7	117.5	33031	32730	30184
CV(%)	15.2	2.9	8.0	0.6	3.6	5.0
Recovery(%)	89.9			92.2		
Matrix effect(%)	93.8			91.4		


*CV %, coefficient of variation = [(SD/M) × 100]; M, mean; SD, standard deviation of M.*

### Stability of Neurounina-1

The stability tests indicated that no significant degradation of neurounina-1 occurred during four freezing and thawing cycles, over a period of 6 h at room temperature and after processing in the self-injector for 52 h at 5°C, as shown in Table 3.

**Table 3:** Stability tests for neurounina-1 in beagle dog plasma.

Estability	Freeze/thaw (4cycles)		Short-term (6 h)		Post-processing (52 h)	
Concentration	(1.5 ng/mL)	(400 ng/mL)	(1.5 ng/mL)	(400 ng/mL)	(1.5 ng/mL)	(400 ng/mL)
Individual value	1.44	401.76	1.37	419.00	1.39	398.17
	1.39	403.30	1.34	426.97	1.62	407.43
	1.35	410.78	1.55	432.58	1.32	416.33
Mean	1.39	405	1.42	426	1.44	407
CV(%)	3.2	1.2	8.0	1.6	10.9	2.2
Accuracy(%)	92.7	101.3	94.7	106.5	96.0	101.8


*CV% = [(SD/M) × 100]; Accuracy % = [(E - T)/T] × 100; CV, coefficient of variation; M, mean; SD, standard deviation of M; E, experimentally determined concentration; T, theoretical concentration.*

### Pharmacokinetic Parameters of Neurounina-1

**FIGURE 3:**
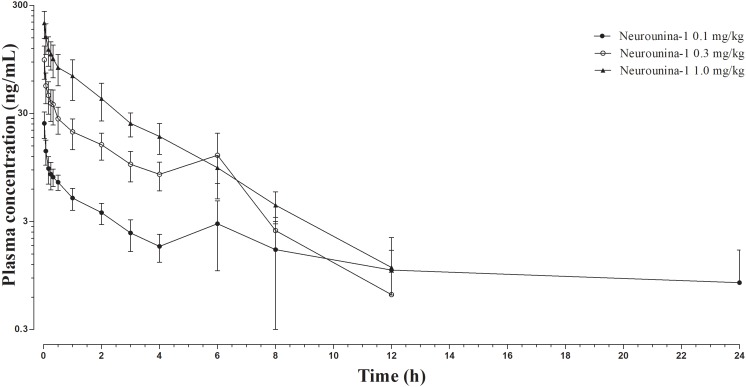
Neurounina-1 plasma concentration vs. time profile obtained after a single intravenous administration of 0.1, 0.3, and 1 mg/kg of neurounina-1 to beagle dogs (*n* = 5). Data are expressed as mean and standard deviation.

The mean neurounina-1 plasma concentration vs. time profiles after a single intravenous dose administration of 0.1, 0.3, and 1 mg/kg neurounina-1 to beagle dogs (*n* = 5) are shown in Figure 3. The obtained neurounina-1 pharmacokinetic parameters are presented in Table 4. The mean AUC_0-tlast_ values were 26.10, 115.81, and 257.28 ng^∗^h/mL following intravenous administration of 0.1, 0.3, and 1 mg/kg, respectively. Linear pharmacokinetics was observed up to 1.0 mg/kg. The neurounina-1 was rapidly eliminated, with mean CL values of 46.24, 47.57, and 69.15 L/h, Vd of 130.31, 154.15, and 210.79 L and t_1/2_ of 2.14, 2.54 and 2.04 h after intravenous administration of 0.1, 0.3 and 1 mg/kg, respectively.

**Table 4:** Pharmacokinetic parameters after a single intravenous administration of 0.1, 0.3, and 1 mg/kg of neurounina-1 to beagle dogs (*n* = 5).

Parameter	Mean	SD	Min	Median	Max	CV%
Neurounina-1 (0.1 mg/kg)						
AUC_0-inf_ (ng^∗^h/mL)	30.10	9.92	23.05	26.44	44.47	32.95
AUC_0-tlast_ (ng^∗^h/mL)	26.10	9.85	18.37	23.00	40.01	37.74
AUC_%extrap_ (%)	4.00	0.82	2.84	3.42	4.68	20.54
C_0_ (ng/mL)	32.47	7.24	24.90	30.64	41.50	22.30
CL (L/h)	46.24	11.30	31.93	47.69	59.17	24.44
Ke (h^-1^)	0.35	0.07	0.29	0.38	0.43	20.39
t_1/2_ (h)	2.02	0.40	1.60	1.67	2.38	20.10
Vd (L)	130.31	17.94	109.65	126.86	151.55	13.76
Neurounina-1 (0.3 mg/kg)						
AUC_0-inf_ (ng^∗^h/mL)	122.42	64.73	35.68	133.20	213.80	52.88
AUC_0-tlast_ (ng^∗^h/mL)	115.81	65.88	28.53	127.76	208.96	56.88
AUC_%extrap_ (%)	6.61	2.14	4.28	4.56	9.63	32.33
C_0_ (ng/mL)	110.54	90.32	44.80	100.43	260.00	81.71
CL (L/h)	47.57	41.36	19.92	35.47	120.24	86.95
Ke (h^-1^)	0.30	0.05	0.22	0.31	0.35	16.45
t_1/2_ (h)	2.36	0.44	1.95	2.16	3.10	18.91
Vd (L)	154.15	112.84	61.82	102.96	338.35	73.20
Neurounina-1 (1 mg/kg)						
AUC_0-inf_ (ng^∗^h/mL)	263.06	192.08	150.98	184.06	601.44	73.02
AUC_0-tlast_ (ng^∗^h/mL)	257.28	190.91	147.67	179.65	593.62	74.20
AUC_%extrap_ (%)	5.78	1.92	3.31	4.55	7.82	33.16
C_0_ (ng/mL)	222.90	170.33	34.70	196.62	490.00	76.42
CL (L/h)	69.15	31.93	17.12	74.91	94.05	46.17
Ke (h^-1^)	0.33	0.04	0.30	0.32	0.40	11.90
t_1/2_ (h)	2.13	0.23	1.75	1.94	2.30	10.66
Vd (L)	210.79	96.51	51.89	224.15	307.70	45.79


*AUC, area under the plasma concentration vs. time curve; t_1/2_, elimination half-life; Ke, elimination rate constant; CL, clearance; Vd, volume of distribution.*

## Discussion

The present study shows, for the first time, a selective and sensitive method for analysis of neurounina-1 in beagle dog plasma using LC-MS/MS. One of the most advantages is that this method does not require deuterated analogs.

The LC-MS/MS method presented a good sensitivity (LLOQ of 1 ng/mL) and permits a high throughput. Furthermore, this method is simple and selective for quantification and pharmacokinetic evaluation of neurounina-1 in beagle dog plasma. Indeed, this method was applied to evaluate the pharmacokinetics of neurounina-1 after a single intravenous administration of three different doses (0.1 mg/kg, 0.3 mg/kg, and 1 mg/kg) to beagle dogs. Linear pharmacokinetics was observed up to 1.0 mg/kg. Nevertheless, the high lipophilicity index of neurounina-1, it showed a low half-life with mean CL values of 46.24, 47.57, and 69.15 L/h, Vd of 130.31, 154.15, and 210.79 L and t_1/2_ of 2.14, 2.54, and 2.04 h after intravenous administration of 0.1, 0.3, and 1 mg/kg, respectively.

Remarkably, prediction analysis revealed that neurounina-1 possesses a high lipophilicity and, thus, a high capability of crossing the blood-brain barrier (BBB), with an estimated log P of 0.87 (n-octanol/water). This aspect is further highlighted by data on the Vd. Indeed, the high Vd values of neurounina-1 confirm the ability of this compound to reach the CNS and support its indication in neurological disorders. As concern, the possible breakdown of BBB after stroke may alter its permeability (Krueger et al., 2017). Therefore, experiments carried out in healthy animals could show lower levels of the drug in the brain tissue. On the other hands, the pharmacokinetic profile determined in beagle dogs correlates with the *in vitro* and *in vivo* therapeutic effect observed in neurons exposed to oxygen glucose deprivation (OGD) and in adult mice subjected to transient occlusion of middle cerebral artery and in neonatal hypoxic mice treated intraperitoneally with neurounina-1 (30 μg/kg) (Cerullo et al., 2018). The dosage used in the present study is in a higher range of that exerting a protective effect in mice cortical neurons exposed to OGD. Furthermore, the most effective dosage of neurounina-1 in an animal model of stroke was 0.03 μg/Kg, administered 3 h after stroke induction (Molinaro et al., 2013), much smaller than the dosage range administered to beagle dogs. A protection, less marked than that obtained with 0.03 μg/Kg neurounina-1, was also seen when neurounina-1 was used at a dose of 0.003 μg/Kg (Molinaro et al., 2013). Notably, the administration of neurounina-1 seemed to be well tolerated by the animals and no sign of behavior modification were observed.

A possible major limitation of this study is the relative short half-life of neurounina-1, around 2 h. For its potential use during the acute stroke episode, intravenous infusion of the drug could be eventually done. However, for the sub-acute or chronic administration of the compound, its fast elimination rate could be an important drawback.

## Conclusion

The developed and validated method to quantify neurounina-1 in beagle dog plasma using LC-MS/MS presented sensitivity and selectivity, thus allowing the rapid and precise determination of the pharmacokinetics of this neuroprotective compound working in the low nanomolar range.

## Ethics Statement

This study was carried out in accordance with the recommendations of the general ethical guidelines established by the Brazilian Society for Laboratory Animal Science (SBCAL). The protocol was approved by the Committee for Ethics in Animal Use – State University of Campinas (CEUA/UNICAMP, protocol n° 3340-1).

## Author Contributions

BS, AC, FeF, FrF, EP, GC, VS, EM, PM, GP, and LA participated the drug development. NA and GM performed the pharmacokinetic and statistical analysis. JR-M and NdF performed the drug administration and blood sampling. GDN was the supervisor. BS, LA, NA, and GM wrote the manuscript. All authors contributed to manuscript revision, read and approved the submitted version.

## Conflict of Interest Statement

The authors declare that the research was conducted in the absence of any commercial or financial relationships that could be construed as a potential conflict of interest.
